# Correlation between twofold left ventricular outflow tract-velocity time integral and stroke volume index in mechanicaly ventilated patients

**DOI:** 10.1186/2197-425X-3-S1-A597

**Published:** 2015-10-01

**Authors:** R Blancas, Ó Martínez González, J Luján, C Martínez Díaz, A Núñez Reiz, D Ballesteros, B López Matamala, C Martín Parra, M Á Alonso Fernández, M Chana

**Affiliations:** Critical Care, Hospital Universitario del Tajo, Aranjuez, Spain; Critical Care, Hospital Universitario Príncipe de Asturias, Alcalá de Henares, Spain; Critical Care, Hospital Clinico San Carlos, Madrid, Spain

## Introduction

Ultrasound is a non-invasive and bedside accessible method of cardiac function monitoring. Cardiac echocardiography has several limitations due to difficulties to obtain an acceptable acoustic window in critical care settings. Left ventricular outflow tract (LVOT) velocity time integral (VTI) can be measured in most critical care patient and is an useful tool for this purpose. Calculations of cardiac output needing aortic valve area are strongly dependent on skills and observers [1]. We hypothesize that twofold LVOT-VTI is an accurate calculation of stroke volume index (SVI).

## Objectives

To assess correlation between SVI and twofold LVOT-VTI.

## Methods

Three ICU from different university hospitals participated in the study. Consecutive patients with invasive haemodynamic monitoring on mechanical ventilation were included. Patient with aortic valve regurgitation or dynamic stenosis on the LVOT were excluded from the study. LVOT-VTI was measured by pulsatile Doppler echocardiography. Five measurements of LVOT-VTI were obtained. Mean and maximum values were recorded. Simultaneously, five measurement of SVI by floating pulmonary artery catheter (PAC) or Pulse Induced Contour Cardiac Output (PiCCO) themodilution methods were obtained and the mean was recorded. Mean and maximum LVOT-VTI were correlated with mean invasive SVI. Statistical analysis: correlation was assessed by Pearson correlation index and intraclass correlation coefficient (ICC).

## Results

Forty paired measurements were recorded in fifteen patients. One patient was excluded from the study due to the difficulty to obtain an adequate acoustic window. Nine patients were monitorized by PiCCO and 6 by PAC. Mean LVOT-VTI correlated better than maximum LVOT-VTI with SVI. Mean LVOT-VTI was 19.21 cm and the mean SVI was 39.34 mL/m2 (Pearson correlation index r = 0.621, p < 0.001; ICC = 0.561, p < 0.001). This correlation worsened when LVOT-VTI was close to 30 cm.

## Conclusions

LVOT-VTI could be useful as a non-invasive simple method to calculate SVI in most of mechanically ventilated patients. Twofold LVOT-VTI seems to be an accurate calculation of SVI, not needing antropometric data. Larger studies are needed to assess factors influencing on erroneous measurements.

**Figure 1:**
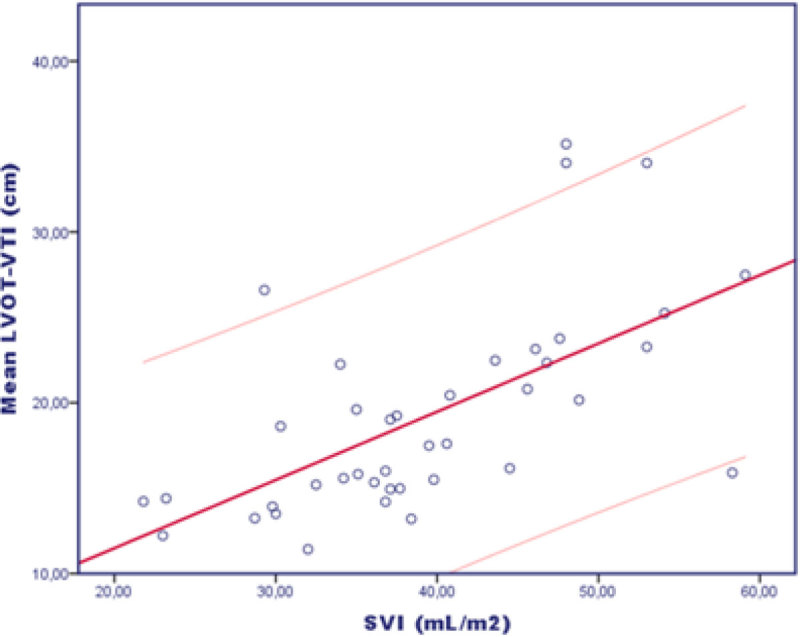
**Correlation of paired data.**
